# Designing Peptide Fossils That Model the Evolution of the Bacterial Ferredoxin Fold

**DOI:** 10.1021/jacsau.5c00863

**Published:** 2025-11-03

**Authors:** Bhanu P. Jagilinki, Ian Campbell, Alexei M. Tyryshkin, Andrew C. Mutter, Jan Siess, Juliana DiGiacomo, Dylan Klein, Saroj Poudel, Paul G. Falkowski, Jonathan J. Silberg, Vikas Nanda

**Affiliations:** † Center for Advanced Biotechnology and Medicine and the Department of Biochemistry and Molecular Biology, Robert Wood Johnson Medical School, 12287 Rutgers University, Piscataway, New Jersey 08854, United States; ‡ Environmental Biophysics and Molecular Ecology Program, Department of Marine and Coastal Sciences and the Department of Earth and Planetary Sciences, 242612 Rutgers University, New Brunswick, New Jersey 08901, United States; § Department of Biosciences, 3990 Rice University, Houston, Texas 77005, United States

**Keywords:** protein evolution, ferredoxins, [4Fe-4S] clusters, catalysis, electron paramagnetic resonance (EPR), electron transfer, iron−sulfur protein, redox reaction, electrochemical titrations

## Abstract

Electron transfer coupled to redox chemistry is at the heart of metabolism. The proteins responsible for moving electrons (protein electron carriers) must have emerged at the origin of life. The small iron–sulfur-binding bacterial ferredoxins were likely among these first proteins. Embedded within the ferredoxin sequence and structure is a symmetry that points to an ancient gene duplication event. Little is understood about the nature of ferredoxins prior to this duplication event or what environmental factors may have driven the selection for more complex forms. The deep-time molecular history of ferredoxins goes back billions of years and cannot be reconstructed by phylogenetic analyses based on amino acid sequences. Here, we use structure-guided protein design to model a fossil half-ferredoxin stage in the evolution of this fold, the semidoxins, and their symmetric full-length counterparts, the symdoxins. Semidoxin designs homodimerize, exhibiting structural, thermodynamic, and electrochemical behaviors in most cases identical to cognate symdoxins. However, the semi- and symdoxin fossil stages behave differently when incorporated into an in vivo electron transfer complementation assay. Both can support bacterial growth dependent on protein expression. Growth rates of bacteria expressing the semidoxins are much more sensitive to oxygen than those of bacteria expressing symdoxins. Motivated by the in vivo functionality of designed semidoxins, we identified putative naturally occurring semidoxins in extant anaerobic microorganisms. This is consistent with the observed in vivo oxygen sensitivity of the semidoxin designs. One natural semidoxin is shown to be folded and redox active. However, it exists as a mixture of monomers and dimers, suggesting a potential connection between semidoxins and even simpler single iron–sulfur cluster-binding peptides.

## Graphical abstract

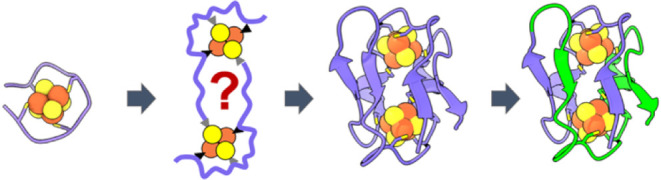

## Introduction

The first proteins that drove the origins of life have long since vanished without any fossil trace. We can surmise that their central function was metabolism—coupling redox energy on the early Earth to biochemical reaction networks.^1–3^ Phylogenetic approaches based solely on sequence data have inherent limitations in resolving deep-time ancestry.^4,5^ By integrating protein structure, cofactor interactions, and geochemical constraints,^6–15^ it is possible to infer ancient evolutionary connections. Diverse approaches converge on a restricted set of protein folds, many of which are associated with transition metal binding that is crucial for supporting redox biochemistry. The bacterial ferredoxin fold was likely among these first redox-active proteins to emerge at the origin of metabolism.^1,16^

There are multiple types of ferredoxins, the bacterial ferredoxins, which are the focus of this study, and the [2Fe-2S] cluster-binding ferredoxins, which are largely found in higher organisms. Several features of bacterial ferredoxins (herein referred to simply as **ferredoxins**) point to their ancient origins. They are small proteins, around 60 amino acids long, that fold as a tandem pair of β-α-β secondary structure elements. They coordinate one or two cubane [4Fe-4S] clusters^17–19^ typically through four highly conserved cysteines per cluster as the first-shell ligands.^20,21^ Ferredoxins typically function as low-potential (−260 to −680 mV vs SHE) single-electron carriers,^22,23^ matching geochemical redox couples abundant in the early Archean Earth and in modern reducing environments.^14,15,24,25^ They can also facilitate two-electron redox reactions, as in the case of nitrogen fixation.^26^ Inorganic iron–sulfur chemistry preceded the origin of proteins and has been suggested to have contributed to prebiotic metabolic reactions.^27–29^ Today, ferredoxins are found in diverse core bioenergetic pathways, from methanogenesis to photosynthesis to aerobic respiration,^14,30–33^ with some species having up to dozens of ferredoxin paralogs.^34^ They shuttle electrons within the cell between donor and acceptor redox proteins or are embedded as domains within larger redox enzymes, acting as wires sometimes spanning several nanometers within a protein.^30,35,36^ The simple fold, redox energetics, and ubiquity in metabolism are consistent with their proposed ancient origin.^34^

Sequence and structural symmetries within ferredoxin suggest an evolutionary trajectory that extends deep into life’s history, perhaps back to a prebiotic–biotic emergence of metabolism. The two β-α-β domains of ferredoxin have sequence homology and are structurally equivalent, related by 2-fold cyclic symmetry, with each domain binding one [4Fe-4S] cluster (Figure 1A).^37^ This architectural symmetry has evolutionary implications, first noted by Eck and Dayhoff in 1966.^38,39^ They proposed a hierarchical trajectory of complexity in multiple stages, starting with a tetrapeptide composed of amino acids present in a presumed early genetic code: aspartate, alanine, serine, and glycine. Repeating the ADSG motif produces a sequence that aligns remarkably well with those of modern ferredoxins. Sequences incorporating amino acids proposed to appear later in evolution, such as cysteine, proline, and valine, improve the similarity to modern ferredoxins significantly.

**Figure 1:**
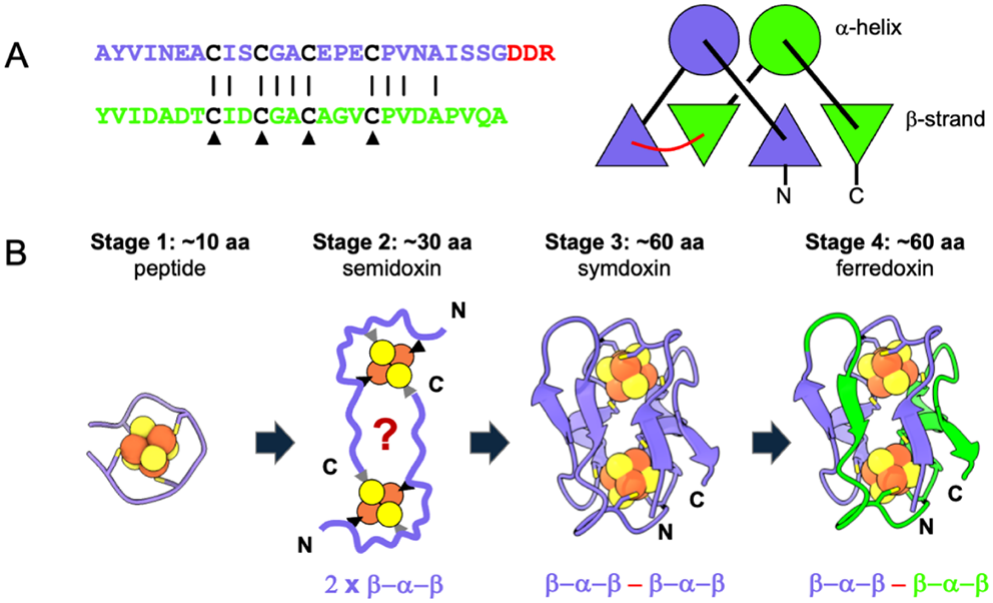
Dayhoff’s hierarchy of ferredoxin evolution. (A) Sequence homology evidence of repeated sequences and 2-fold structural symmetry when aligning two regions of a single natural ferredoxin (sequence and aligned domains of *C. acidurici* ferredoxin.^53^ (B) Ferredoxin evolution based on the original Dayhoff proposal.^39^ Little is known about the structure and function of the first two stages. Stage 1 is modeled from the [4Fe-4S] binding peptide ambidoxin.^42^ Duplication and fusion of two semidoxins likely generated full-length symdoxins that diversified into extant ferredoxins.

Here, we adapt the Dayhoff hierarchy to include cysteine at the earliest stage of evolution, allowing for the selection of metal-binding activity and redox function. It has been shown that cysteine can be produced under prebiotically plausible conditions from nitrile analogs of serine and pathways for subsequent oligopeptide synthesis.^40^ Short cysteine-containing oligopeptides can bind iron–sulfur clusters in defined, redox-stable configurations.^41,42^ As such, it is plausible that **Stage 1** would include short metal-binding peptides that could coordinate iron–sulfur centers with cysteine and facilitate single low-potential electron transfers. At **Stage 2**, longer sequences emerged by the repetition of a short peptide motif, as Dayhoff suggested. These might have adopted a β-α-β fold and associated with a homodimer with two clusters. Then, at **Stage 3**, a duplication event could produce (β-α-β)-(β-α-β) ferredoxins of around 60 residues. Finally, in **Stage 4**, diversification of the N- and C-terminal domains would lead to the evolution of extant ferredoxin paralogs (Figure 1B). The Dayhoff framework can be used to develop concrete, testable models for recapitulating the deep-time evolution of the ferredoxin fold.

Candidates for Stage 1 molecules have been proposed and studied. Short peptides with one or multiple cysteines have been demonstrated to bind [4Fe-4S] clusters and, in some cases, reversibly cycle between oxidized +2 and reduced +1 states.^43–47^ These synthetic peptides can function as electron carriers, driving the formation of a pH gradient in protocell models.^48^ The chemical activity and incipient functionality of these peptides support a plausible connection between genetically encoded iron–sulfur proteins and spontaneously assembling metallopeptides during the emergence of metabolism.^49–52^

Stage 3 of Dayhoff’s hierarchy posits a fully symmetric, single-chain ancestor of extant ferredoxins. This stage has been modeled using ancestral sequence reconstruction, indicating increasing sequence identity between N- and C-terminal halves at branches approaching the root of the ferredoxin tree.^38^ Computational structure-guided designs of symmetric ferredoxins (referred herein as a **symdoxin**) could express and assemble with [4Fe-4S] clusters in vivo, and they could function as electron carriers in an engineered pathway within a cell.^54,55^ Symdoxins represent a potential evolutionary bridge between metallopeptides and modern ferredoxins.

Bridging the first and third stages is a half-ferredoxin, which we refer to here as a “**semidoxin**.” We know very little about this putative second stage. Previously, a fragment of a natural semidoxin that corresponds to a semidoxin was shown to bind an iron–sulfur cluster and could be reversibly oxidized and reduced between the +2 and +1 oxidation states.^43^ However, we know little about the actual structure or functional relevance of such short proteins. Semidoxins may have been monomeric, binding a single [4Fe-4S] cluster, or they may have existed as obligate homodimers. What selective pressures drove the transition between stages 2 and 3 of Dayhoff’s hierarchy? What advantage did the fusion of two semidoxins into a single symdoxin gene confer? The covalent linkage of semidoxins may have increased their thermodynamic stability and/or improved resilience to redox-excited states over multiple oxidation/reduction cycles. Alternatively, the transition may have been neutral, with functional advantages evident only after subsequent diversification.^56,57^ To explore these questions, we designed and characterized cognate semidoxins and symdoxins^54^ that simulate the transition from Stage 2 to 3 of Dayhoff’s hierarchy.

Here, we extend an approach previously applied to the Stage 3 to 4 transition in the Dayhoff hierarchy.^54^ Previously, the consensus-designed ferredoxins, ANC and SNC, were generated by selecting the most frequent amino acid at each position in a multiple sequence alignment. Consensus design, often applied to domain-repeat proteins, highlights conserved structural features while minimizing lineage-specific traits.^58^ The ANC design was constructed from a broad set of ferredoxin sequences, including domains embedded in larger proteins, whereas the SNC was based on a smaller set of soluble ferredoxins. These designs were then modified to generate cognate symdoxins—ANN, ACC, SNN, and SCC—by repeating either the N- or C-terminal half. ANC, ACC, and all of the symdoxins adopted ferredoxin-like structures and activities, binding two [4Fe–4S] clusters with reversible redox cycling and midpoints around −400 to −500 mV vs SHE. Moreover, SCC could transfer electrons between donor and acceptor oxidoreductases in living cells, demonstrating that symdoxins are plausible intermediates in ferredoxin evolution. Here, we extend this system to examine four semidoxins designed as the unlinked counterparts of their cognate symdoxins. We test whether these semidoxins are structured, redox active, and capable of electron transfer in vivo, and we compare these features to those of the full-length symdoxins.

## Results and Discussion

### Semidoxin Fusion Maintains Metal Coordination and Structure

Previously designed symdoxins ACC, ANN, SCC, and SNN were converted into four cognate semidoxins, AN, AC, SN, and SC (28 aa long), by using only half the sequence and adding a Trp-Gly to facilitate the measurement of peptide concentration:

**ANN:AYIITEKCIGCGKCARVCPVDAISGE**VKK**AYIITEKCIGCGKCARVCPVDAISGE**

**AN:AYIITEKCIGCGKCARVCPVDAISGE**WG

Sequences for AC, SN, and SC semidoxins were generated using the same strategy and are reported in the Materials and Methods.

Semidoxins were produced via solid-phase synthesis, purified by reverse-phase HPLC, and confirmed using MALDI-TOF mass spectrometry (Figure S1). A chemical reconstitution step was required for iron–sulfur cluster incorporation using previously published protocols.^46^ The reconstituted holo-semidoxins appeared reddish-brown, typical of Fe–S proteins.^59,60^ UV–visible spectra of the semidoxins exhibited the characteristic 400 nm peak (Figure S10A), which disappeared upon reduction with sodium dithionite (NaDt) (Figure S10A inset). These spectral characteristics are consistent with those observed in reported [4Fe-4S] sites in ferredoxins.^54,61,62^

Size exclusion chromatography under anaerobic conditions showed that all four semidoxins eluted as a single species, with protein absorption monitored at 280 nm. The protein peaks coincided with iron–sulfur binding monitored at 415 nm (Figure S10B). The electronic structure of these clusters was examined by continuous wave electron paramagnetic resonance (cw-EPR) spectroscopy, confirming [4Fe-4S] clusters in the reduced +1 state (Figure 2A).^63^ The presence of weak and broader signals between 320 and 340 mT is consistent with spin coupling of two [4Fe-4S]^1+^ clusters separated by ∼8 Å.^64^ This feature was also observed in symdoxin spectra.^54^ The absence of signals corresponding to [3Fe-4S] in samples without NaDt indicated close to 100% [4Fe-4S] reconstitution assembly. Together, these observations indicate that each semidoxin binds two [4Fe-4S] clusters. Such binding would implicate semidoxins assembling as homodimers. The putative iron–sulfur cluster-bound, homodimeric forms of semidoxins are designated as 2AN, 2AC, 2SN, and 2SC.

**Figure 2:**
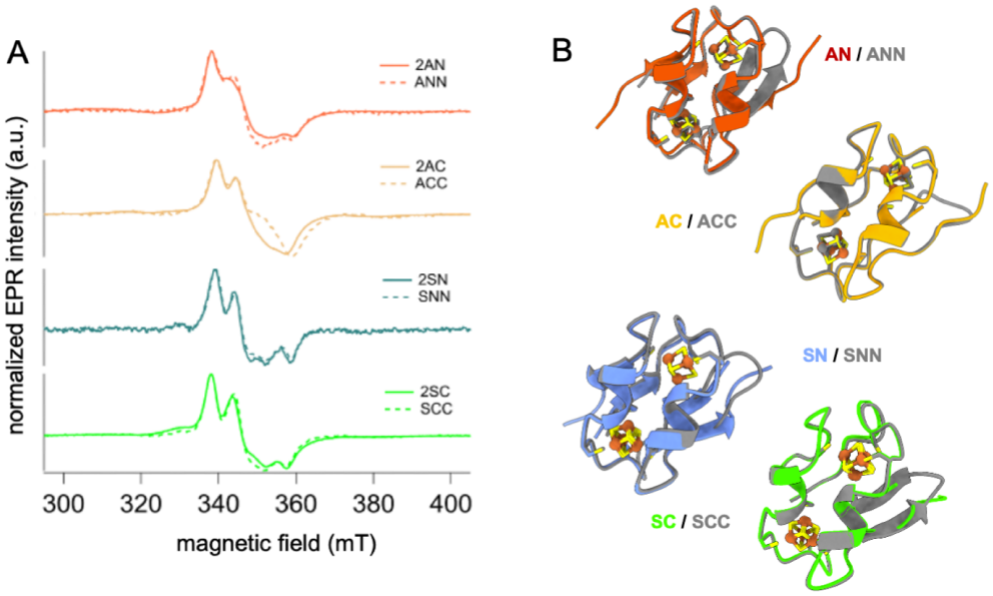
Structural similarity of cognate semi- and symdoxins. (**A**) X-band CW-EPR spectra of semidoxins and cognate symdoxins: X-band CW-EPR spectra of semidoxins (solid lines) and cognate symdoxins (broken lines) confirm reduced [4Fe-4S]^+1^ clusters with similar electronic structures. (B) Boltz-1 models show structural identity around cluster sites.

The EPR spectral features of semidoxins were compared with symdoxin spectra.^54^ Despite variations in fine structure between spectra of different designs, semidoxin spectra matched those of their cognate symdoxin (Figure 2A). Given the sensitivity of the electronic spectra to the structure and dynamics of the primary ligands and secondary coordination spheres,^65^ this observation indicates significant structural similarity near the [4Fe-4S] cluster coordination sites between cognate pairs. The exception is the 2AC/ACC pair, which shows different EPR features. This design has a histidine near the first-shell ligands, which was previously shown to affect redox energetics in ACC.^54^

Boltz-1 models^66,67^ of the designs support high structural similarity between cognate pairs (Figure 2B), with sub-Angstrom deviations in the alignments of backbone and side-chain groups around the metal site. The structural deviations are <0.3 Å RMSD, and the pLDDT confidence scores are >0.9 throughout the protein, except for the chain termini and in the central turn of symdoxins, where their sequences diverge. No frank structural differences are noted between the models of ACC and 2AC that explain EPR spectra differences.

The homodimeric assembly of the designed semidoxins allows them to bind two 4Fe-4S clusters per dimeric complex. Ancestral semidoxins may have formed similar dimeric complexes, suggesting that redox features, such as redox potential tuning through cluster–cluster interactions and multielectron transfer reactions, may have already been possible at Stage 2 in the Dayhoff trajectory.

### Semidoxin Fusion Maintains Structural Stability

Fusion of two semidoxins by a short linker might lead to enhanced stability of the folded state by increasing the effective concentration of the two domains. There is precedence for this in engineered single-chain analogs of homodimeric Arc repressor, where increasing linker length leads to decreased stability.^68^ We hypothesized that the need for increased stability could have been a selective pressure for the evolution of symdoxins. To test this idea, we measured the unfolding energetics of all four cognate pairs by thermal denaturation, monitoring the structure by far-UV circular dichroism spectroscopy. The denaturation profiles of the different semidoxin designs vary in the number of transitions, transition cooperativity, and transition temperatures. In contrast, the cognate symdoxin/semidoxin profiles are very similar (Figure 3). Thus, fusing semidoxins does not enhance stability. This analysis does not represent a complete thermodynamic picture as we were not able to probe the refolding energetics due to the chemical instability of uncoordinated [4Fe-4S] clusters arising from competing iron-sulfide precipitation reactions.^69^ Metal–ligand interactions may dominate folding energetics, and the primary differences in stability may manifest in the apo-proteins. The observed changes in stability between the designed semidoxins and symdoxins are minimal. If this were also the case for ancestral semidoxins, then biophysical stability alone would have been an unlikely driver of fusion.

**Figure 3:**
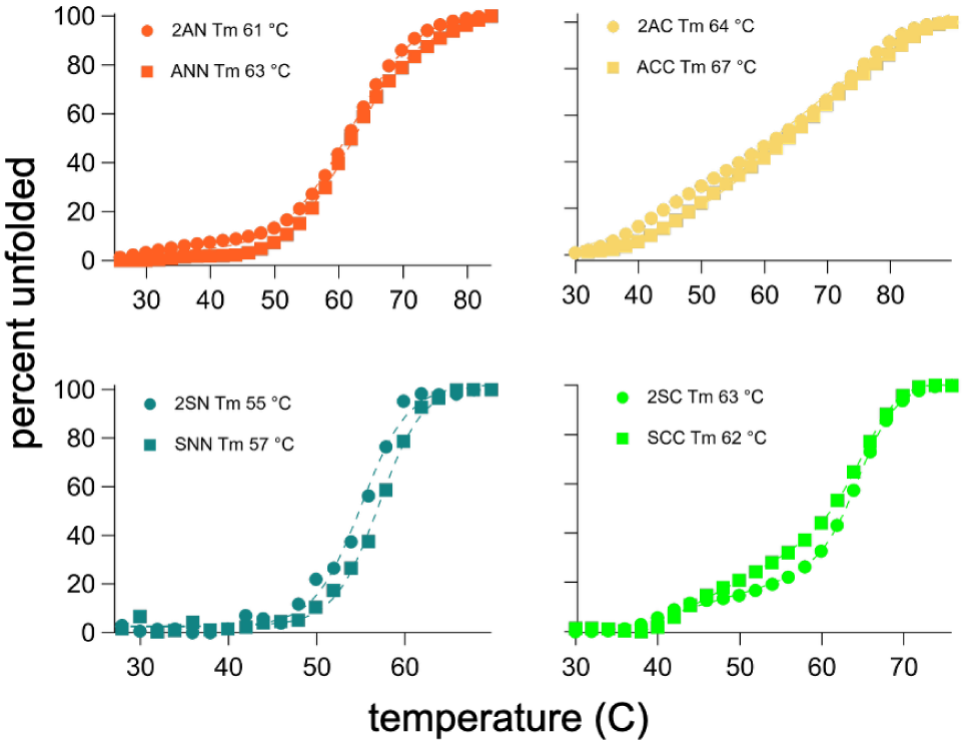
Semidoxin vs symdoxin stability and dynamics The thermal denaturation profiles monitored by CD (208 nm) of semidoxins and cognate symdoxins are matched. Fitted melting temperatures (Tm) are listed in the panels. See Figure S9 for CD spectra.

### Semidoxin Fusion Maintains Redox Energetics

Redox transitions in protein active sites drive reorganization in response to electronic changes in the metal and local coordination sphere. The stability of the protein fold and metal coordination during reorganization can affect redox energetics and is likely under evolutionary selection.^70^ The similarities in the structure and stability described so far have been observed in the ferredoxin resting state. To test if the evolutionary transition from semidoxin to symdoxin may have been driven by altered redox energetics, chronoamperometric titrations were performed using the optically transparent thin-layer electrochemical (OTTLE) cell technique with a honeycomb gold electrode.^71–73^ All titrations reached complete reduction, as monitored spectrophotometrically at 430 nm. Titrations were reversible in oxidation and reduction directions (Figure 4). The semidoxin midpoint potentials closely matched the published E_m_s of the cognate symdoxins.^54^ E_m_s were small, with the largest for 2AN/ANN being 17 mV, which is less than kT (∼25 mV at 298 K). The similarity of the semidoxin and symdoxin electrochemical properties suggests that both semidoxin and symdoxin topologies accommodate the resting and excited oxidation states of the two 4Fe-4S clusters.

**Figure 4:**
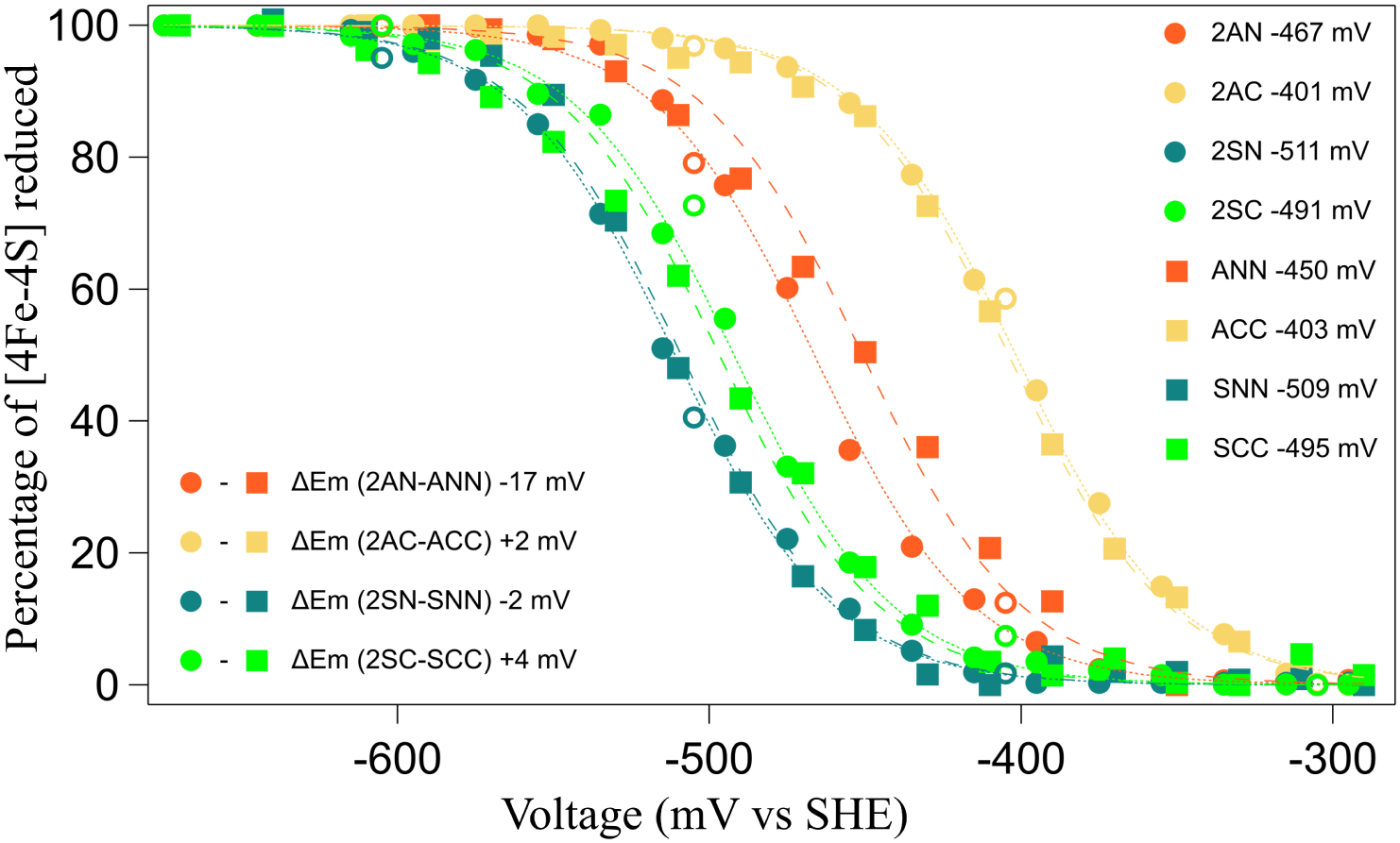
Symdoxin and semidoxin redox energetics are matched. Potentiometric titrations of semidoxins (circles) versus symdoxins (squares): Titrations were fit to the Nernst equation, where *n* = 1.^74^ Closed circles represent points during the reduction cycle, while open circles represent the oxidation phase. All titrations were conducted using an Ag/AgCl reference electrode (Pine Research) and a Gold Honeycomb spectro-electrochemical electrode card (Pine Research), which served a dual purpose as both the counter and working electrodes.

### Semidoxins are Sensitive to Oxygen In Vivo

We previously found that symdoxins can support cellular electron transfer in a synthetic pathway within *Escherichia coli* under microaerophilic conditions (pO_2_ = 0.2%)^54,55^ (Figure 5A). Here, we find that semidoxins are also sensitive to oxygen and can rescue function under microaerophilic conditions, but at significantly lower levels than their cognate symdoxins. In this strain, cell growth only occurs in a medium containing sulfate as the sulfur source when a ferredoxin supports electron transfer from ferredoxin-NADH reductase (FNR) to sulfite reductase (SIR). With multiple symdoxins, cell growth was rescued, as observed with other extant ferredoxins. Given the similarities in the structure, stability, and electrochemical properties of cognate symdoxin and semidoxin pairs, we hypothesized that semidoxins may also support electron transfer from FNR to SIR.

**Figure 5:**
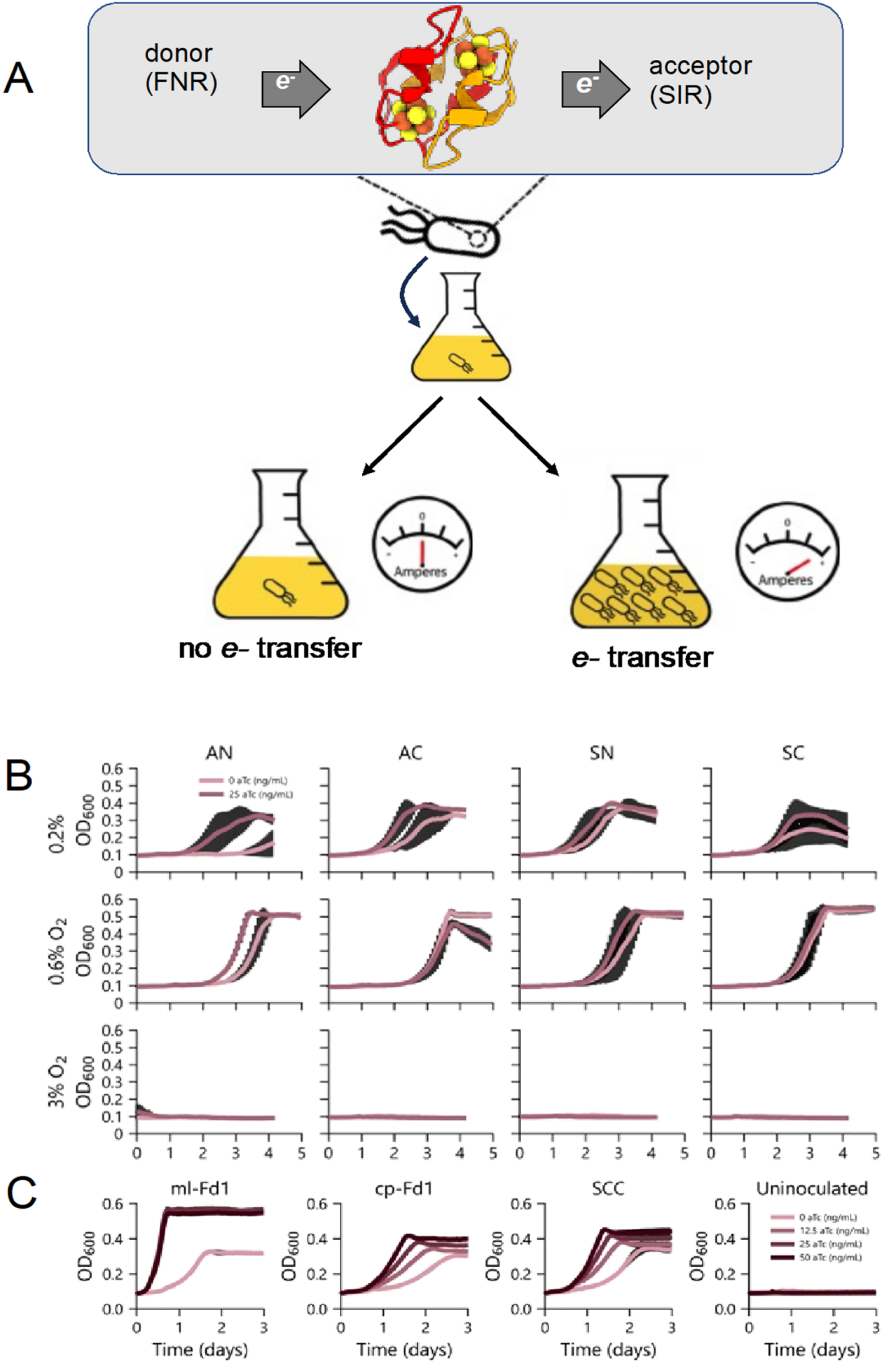
Semidoxin function in vivo. (A) A three-component complementation assay was used to examine function in vivo. Semidoxin expression was induced in the presence of electron donor FNR and electron acceptor SIR. Functional electron transfer by semidoxin would allow cells to grow on a sulfur source. (B) Growth curves of induced/uninduced semidoxin show its ability to shuttle electrons in vivo. 3% oxygen concentration completely inhibited growth. Growth coupled with the induction of semidoxin was observed at very low oxygen tension. These experiments involved multiple biological replicates, with the shaded regions representing the standard deviation. (C) Positive controls ml-Fd-1 (a 2Fe-2S ferredoxin from *Mastigocladus* laminosus) and cp-Fd1 (a 4Fe-4S ferredoxin from *Clostridium pasteurianum*) and the SCC (symdoxin) were grown with 0.2% oxygen and are included for comparison. The right panel (uninoculated) shows the negative control.

Biological electron transfer assays were performed following established protocols in a Tecan plate reader with three replicates^54,55,75^ (see Methods for details). The positive control cyanobacterial 2Fe-2S ferredoxin strongly rescued growth upon induction, exhibiting both reduced lag time and a faster growth rate (Figure 5B). Similarly, a clostridial 4Fe-4S bacterial ferredoxin rescued growth upon expression. The SCC symdoxin rescued growth in an expression-level-dependent manner. In our previous work, under stringent removal of sulfide from cells, only this symdoxin showed growth rescue.^54^

For the semidoxins, we examined growth under constant oxygen levels at three concentrations: 3%, 0.6%, and 0.2%. At 6% oxygen, no growth rescue was observed upon induction. Under intermediate 0.6% oxygen, rescue was observed in both induced and uninduced semidoxin expression conditions, potentially due to cell utilization of trace amounts of sulfide in the culture. Only at microaerophilic concentrations was any rescue observed upon the induction of semidoxin expression. The growth enhancements were small as compared to positive controls and SCC (Figure 5C).

To characterize the intrinsic oxygen sensitivity of the cognate symdoxin and semidoxin pairs, the decay of the iron–sulfur complex UV absorption band at 433 nm was monitored continuously for 10 h after exposing the sample to ambient atmosphere. The fitted half-lives were similar (120 min for 2SN, 150 min for ANN, respectively) (Figure S2). EPR spectra confirm the loss of [4Fe-4S]^2+^ upon exposure to oxygen, leaving a g = 2.01 signal corresponding to an oxidized [3Fe-4S]^1+^ cluster.^76^ This is consistent with reported pathways for cluster conversion by oxygen in FNR^77^ and ferredoxin.^78^ Chemical stability in vitro does not sufficiently account for the functional differences between semidoxin and symdoxin in vivo.

### Extant Semidoxins Persist in Anaerobic Genomes

While the semidoxin designs could function in an engineered pathway in *E. coli*, they do not appear optimized for electron transfer in this facultative anaerobic organism. Natural low-potential semidoxins likely evolved to specifically bind target donors and acceptors and match their redox energetics. Given the suggested functionality of designed semidoxins, we sought to investigate if natural counterparts can still be found in modern environments that are considered proxies for early Earth.

Extant anaerobic microorganisms can be useful proxies for paleoenvironments,^79^ and we hypothesized that if semidoxins still exist, they would most likely be found in organisms from anaerobic niches. To evaluate this hypothesis, we examined a dataset^34^ of [4Fe-4S] binding ferredoxins extracted based on InterPro sequence signatures^80^ from 7079 predominantly bacterial, complete genomes. A small number of short sequences were found (Figure S3), with nearly all occurring in genomes of anaerobic microorganisms (Figure 6A). Given the independent probabilities of microbes in the dataset being anaerobic and for ferredoxins to be <60 aa long, the observed abundance is approximately 10-fold higher than random expectation. Very short, semidoxin-like protein sequences are rare in nature, and these are almost exclusively found in organisms growing in anaerobic niches.

**Figure 6:**
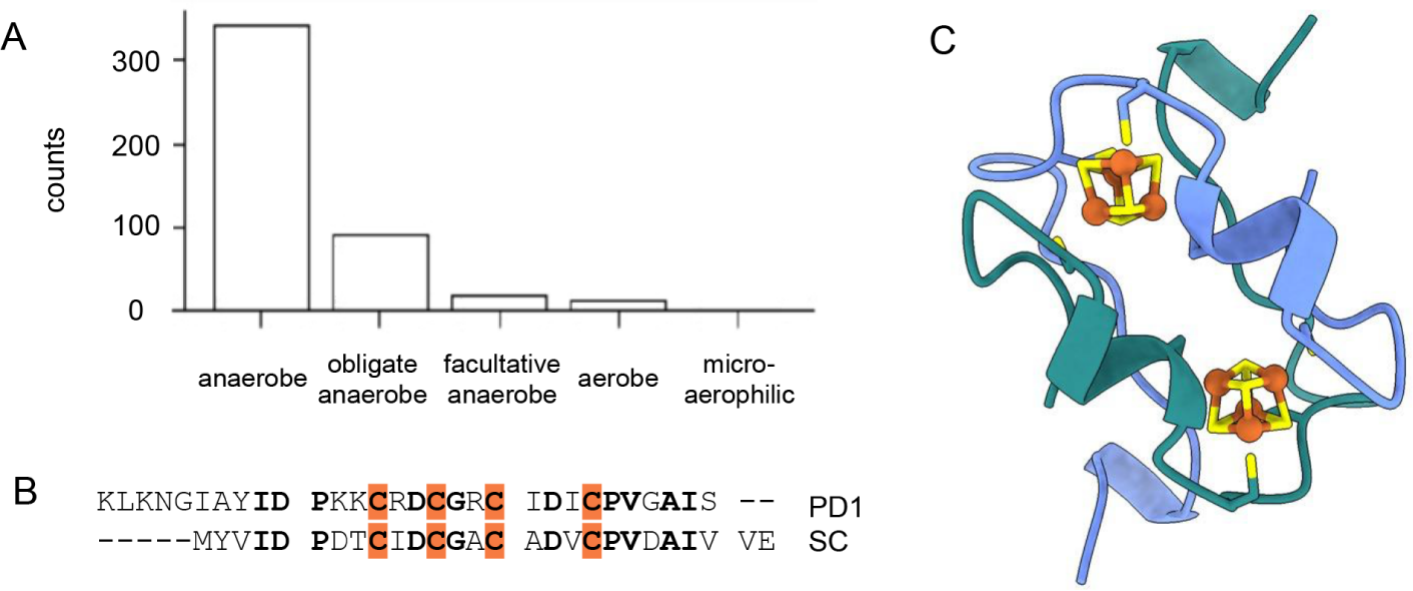
Searching for extant semidoxins. (A) Short (<60aa) ferredoxins are primarily found in anaerobic microorganisms—annotated based on JGI GOLD oxygen requirement.^81^ (B) PD1 from Thermoanaerobacter shares sequence homology with SC, including first-shell cysteine ligands. (C) Boltz-1 model of a PD1 homodimer shows a typical ferredoxin fold.

Most short putative ferredoxins contain two sequence motifs that are predicted to coordinate the [4Fe-4S] clusters. Just a handful, 55 sequences, met the more stringent criteria of being close in size to semidoxins (∼30 amino acids long) and comprising one [4Fe-4S] cluster binding site. One case, found in multiple species of the genus Thermoanaerobacter, closely resembles the SC semidoxin sequence (Figure 6B). We designate this protein here as protodoxin-1 (**PD1**) because of its kinship to Dayhoff’s hypothetical short proto-ferredoxin. Boltz-1 models the PD1 homodimer with a 2x (β–α–β) fold (Figure 6C).

PD1 was produced by solid-phase peptide synthesis, and the product was verified by mass spectrometry (Figure S4). When reconstituted with iron–sulfur species, as described for the designed semidoxins, PD1 presented a UV–visible spectrum that is consistent with those of ferredoxins, with an absorption band around 400 nm (Figure S5). Electrochemical titrations showed reversible oxidation–reduction with a midpoint of −438 mV (pH 7), typical of bacterial ferredoxins (Figure S6).

Unlike the designed semidoxins, at pH 7, PD1 binds iron–sulfur as a ∼2:1 mixture of monomeric and dimeric forms, with both oligomeric forms showing iron–sulfur absorption (Figure 7A). The CW-EPR spectrum of PD1 is distinct from that of the designed semidoxins (Figure 7B). Its spectrum can be reconstructed by the linear combination of a simulated single [4Fe-4S] cluster^42^ and the coupled 2­[4Fe-4S] cluster spectrum of 2SC, assuming a 2:1 ratio of monomeric and dimeric species (Figure 7B), consistent with the FPLC chromatogram. Models of the monomeric holo-PD1 directly obtained from Boltz-1 were not consistent with the tetrahedral coordination of the cluster inferred from EPR. However, energetic remodeling of these predictions using AMBER did produce a plausible structure of the monomer (Figure 7C). The monomer–dimer plasticity of PD1 suggests a functional path from a monomeric Stage 1 metallopeptide to a homodimeric Stage 2 semidoxin. These findings provide evidence that semidoxin genes observed in anaerobes encode peptides that can form low-potential metalloproteins.

**Figure 7:**
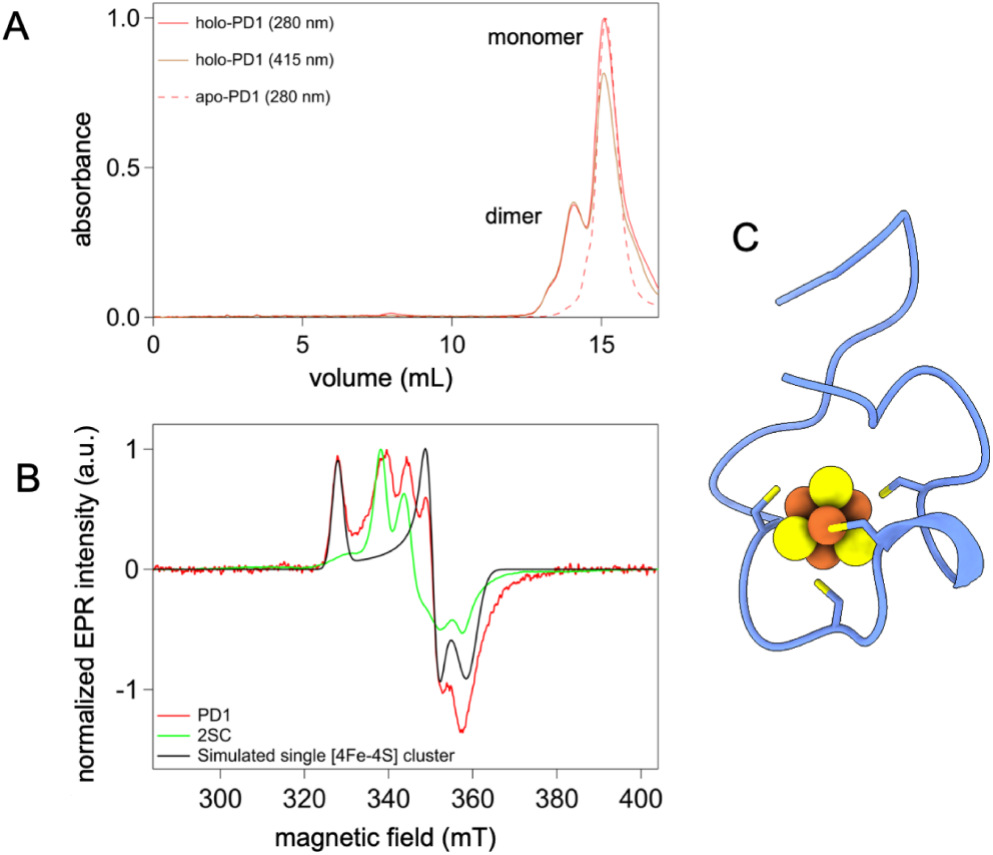
Holo-PD1 is a mixture of monomers and dimers. (A) FPLC chromatogram of holo-PD1 indicates monomer and dimer, as indicated by the 415 nm trace (brown). The apo-PD1, as a monomer, is shown in broken lines (red). (B) CW-EPR analysis confirms the presence of both single [4Fe-4S] and a coupled [4Fe-4S] species in PD1 at pH 7. 2SC spectrum and simulation of single [4Fe-4S] species are overlaid for comparison. (C) Energy-minimized model of the holo-PD1 monomer.

### Emerging Symdoxins

To explore whether PD1 is proceeding along the Dayhoff hierarchy, we searched genomic sequences for potential cognate PD1 symdoxins comprising two repeats of the sequence. However, no clear examples of a PD1–PD1 fusion were identified. Instead, PD1 was most closely related to a larger putative [4Fe-4S] binding protein from Thermoanaerobacter as the second half of a C-terminal ferredoxin domain (Figure S7). If new semidoxins are regenerated from the fission of larger proteins, then semidoxin fusion into symdoxin events may also be occurring in extant genomes.

To directly search for extant symdoxins, ferredoxin sequences from the dataset were split into N- and C-terminal halves and compared for sequence similarity. Phylogenetic analysis of these domains shows that the majority of N vs C-halves from the same ferredoxin are dissimilar (an average cophenetic distance of 4.2). However, a few ferredoxins had high identities across the two halves, with a cophenetic distance <1 (Figure 8), indicating they likely underwent recent gene duplication. Boltz-1 models of these proteins show high structural symmetry as well.

**Figure 8:**
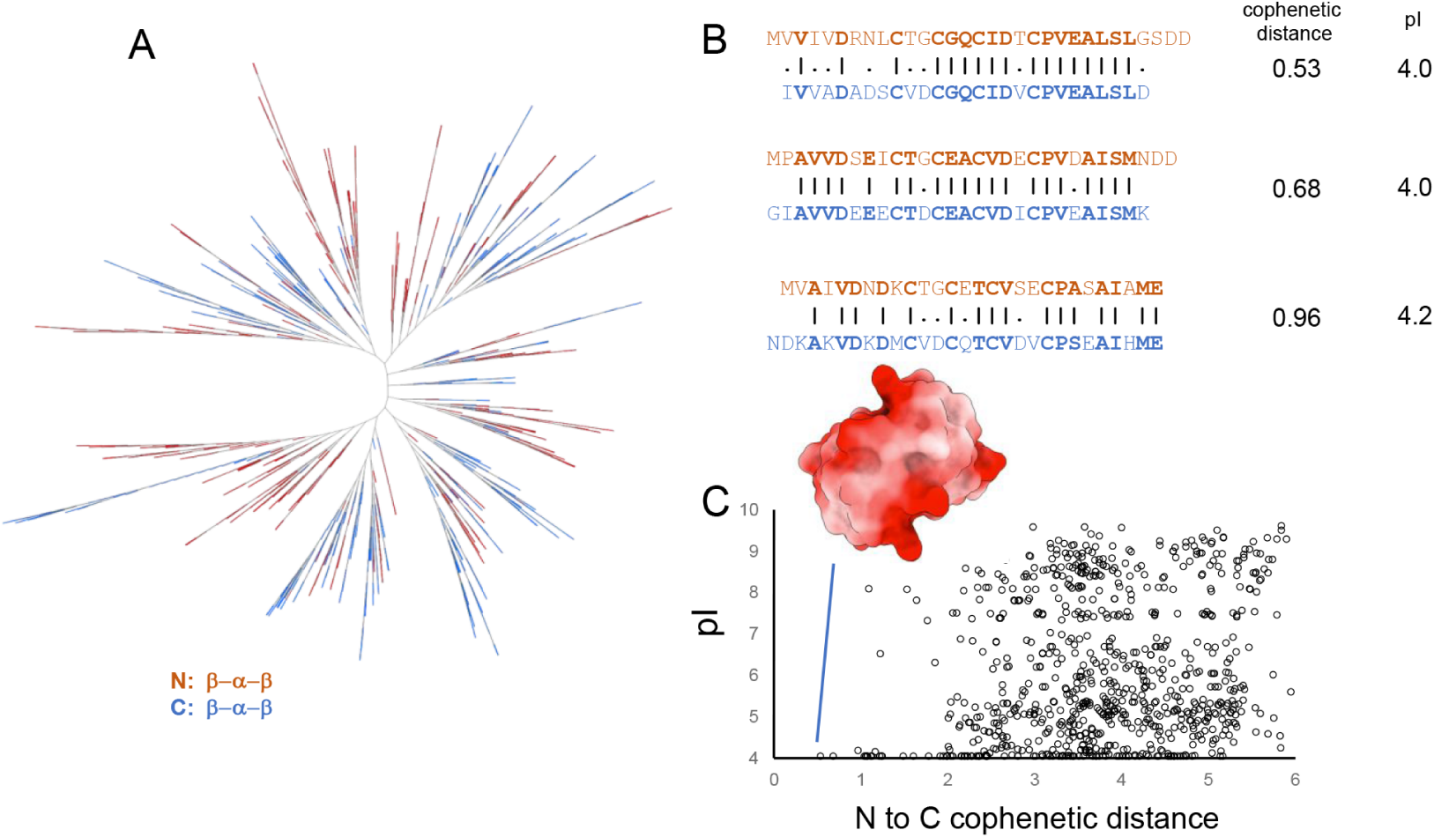
Symmetric ferredoxins (A) Split ferredoxin dendrogram. (B) Alignment of symmetric ferredoxin N (red) and C (blue) domains and corresponding cophenetic distance and pI from *Methanohalobium evestigatum*, *Methanomethylovorans hollandica* and *Methanoregula formicica* (C) For high-symmetry ferredoxins (cophenetic distance <2), 24/30 sequences have pIs ∼4. The electrostatic potential surface for *M. evistigatum* is highly anionic (mean −6.5 kcal/mol·*e*).

There is a correlation between the isoelectric point of the protein and cophenetic distance, with internally symmetric ferredoxins having a high number of acidic residues on the surface. Many redox partners of ferredoxins have patches of basic amino acids that allow them to interact electrostatically.^54,82–84^ We speculate that the low isoelectric point of putative new ferredoxins allows them to function as generic electron shuttles. Subsequent evolutionary diversification of the N- and C-halves would facilitate more specific protein–protein interactions.^85^ In previous work on symdoxins, the acidic SNN and SCC designs were more functional in vivo than those with more neutral isoelectric points. Local electrostatics play a primary role in determining ferredoxin redox energetics.^86^ The high density of negatively charged groups in acidic sequences found in symmetric ferredoxins would lower their redox potential, making them more reducing. This characteristic may also have been present in ancient ferredoxins, where lower potentials would have been better matched with environmental redox couples.^13^

## Conclusions

Ferredoxin is one of many extant folds that exhibit internal pseudosymmetry, strongly indicating evolution from smaller fragments. This work explored the question of whether the ancestral fragments of ferredoxin inherently oligomerized or evolved multimerization separately. The similar optical and electronic spectral features, biophysical properties, and redox energetics of semidoxins and symdoxins indicate an evolutionary trajectory where Stage 2 proteins likely evolved in the context of a homodimeric structure. This is consistent with similar studies of other folds like the 3-fold centrosymmetric β-trefoil, where designed putative ancestral fragments associated as trimers;^87^ for β-propeller pentamers, where truncated versions oligomerized;^88^ and other cases.^89–91^ As noted in many of these studies, the evolution of fragments in the context of a symmetric oligomer ensures foldability at every step along the evolutionary trajectory.^92–94^ In their original work, Eck and Dayhoff suggested that the structural perturbation upon a semidoxin fusion was minimal: “In the three-dimensional structure, the effect of this change was to attach the two shorter chains end-to-end. They must have already been in a configuration that was only moderately disturbed by this new constraint. The attachment was an improvement but not a radical change.” As such, the bacterial ferredoxin fold did not emerge because of a direct selection for symmetry but rather because symmetry provided an evolutionary path where oligomeric intermediates readily and rapidly folded.

Using designed semi- and symdoxins as models of the Dayhoff trajectory, we find the transition between Stages 2 and 3 provides negligible chemical or biophysical advantage to the ferredoxin fold. The differing oxygen tolerance in vivo hints at a possible functional advantage in electron transfer, but oxygen-mediated cellular processes, such as shifts in global redox poise, switching between Isc and Suf systems for Fe–S cluster biogenesis,^95^ and other systemic changes in proteostasis, can complicate this interpretation. The designed proteins do demonstrate that semidoxins can be active in vitro and functional in vivo and that a Stage 2 homodimer is an evolutionarily plausible intermediate.

The electronic spectra of the natural semidoxin PD1, showing both single and di-cluster forms, suggest that it may have existed between the shortest metallopeptides (Stage 1) and the obligate dimeric semidoxins (Stage 2). Extant semidoxins appear to be rare and, thus far, are only found in thermophilic anaerobes, though their functions remain untested. Fundamental questions remain as to whether semidoxins like PD1 are the result of the fission of larger oxidoreductases or holdovers from an ancestral origin. If the latter is the case, this would support a transition from Stage 1 to Stage 2, where semidoxins that bind only one cluster were intermediary states of ferredoxin evolution in an early Earth reducing environment.

## Materials and Methods

### Semidoxin Design Selection

The symdoxins were manually generated from the single-chain symdoxin designs by selecting only the N-terminal half and omitting the C-terminal region from the linker region.^54^ For designs AN and AC, sequences downstream of the linker VKK region were excluded, while for SN and SC, sequences downstream of DKA were omitted. Additionally, a Trp residue was appended to the C-terminal end for spectroscopic analysis, and a Gly residue was included as a derivative of the resin (Fmoc_gly_wang) used in peptide synthesis. The resulting sequences for the four semidoxin designs spanning 28 amino acids are provided below:

**AN:**AYIITEKCIGCGKCARVCPVDAISGEWG

**AC:** HVIDQDKCIKCGACIEACPVDAIIKAWG

**SN:** AYVINDACIACGACVEECPVDAISEGWG

**SC:** YVIDPDTCIDCGACADVCPVDAIVVEWG

The natural semidoxin from *Thermoanaerobacter* was synthesized in the following sequence:

**PD1:** KLKNGIAYIDPKKCRDCGRCIDICPVGAIS

Symdoxin sequences are as reported in ref. 54.

**ANN:**AYIITEKCIGCGKCARVCPVDAISGEVKKAYIITEKCIGCGKCARVCPVDAISG

**ACC:** HVIDQDKCIKCGACIEACPVDAIIKAEVKKHVIDQDKCIKCGACIEACPVDAIIKA

**SNN:** AYVINDACIACGACVEECPVDAISEGDKAAYVINDACIACGACVEECPVDAISEG

**SCC:** YVIDPDTCIDCGACADVCPVDAIVVEDKAYVIDPDTCIDCGACADVCPVDAIVVE

### Peptide Synthesis

Peptides were synthesized via solid-phase synthesis using a Liberty Blue (CEM) peptide synthesizer.^96,97^ Fmoc_gly_wang resin served as the initial scaffold for all synthesized peptides.^98^ The protected amino acids used for synthesis were purchased from CEM. *N*, *N*-Dimethylformamide (DMF) was used as the main solvent, and 20% (v/v) piperidine in DMF was used as the deprotectant. For coupling *N*, *N*’-Diisopropylcarbodiimide (DIC) and Oxyma in DMF were used.^99,100^ Post synthesis, the resin and protecting groups were cleaved using a cocktail of trifluoroacetic acid (TFA)/phenol/water/triisopropylsilane (TIPS) in the ratio of 88:5:5:2.^101^ Following a 4-h incubation, cleaved peptides were precipitated with cold diethyl ether, washed, and dissolved in 0.1% TFA in water. Reverse-phase HPLC was performed, and fractions were collected by monitoring the absorbance at 280 nm.

PD1 peptide was purchased commercially from GenScript. The mass of the apo-PD1 was determined using MALDI-TOF mass spectrometry (Figure S4). Considering the theoretical pI of PD1 (∼8.87), chemical reconstitution with Fe–S salts was carried out at pH 7.

### Mass Spectrometry

Fractions obtained from reversed-phase HPLC were individually collected and analyzed using a mass spectrometer (Applied Biosystems, 4800 MALDI TOF/TOF Analyzer). For MS sample preparation, 1 μL of the HPLC sample was mixed with 1 μL of alpha-cyano-4-hydroxycinnamic acid (CHCA from Sigma), and 1 μL of the mixture was added to the MALDI plate and left to air-dry. The air-dried plate was then analyzed using MS, and the corresponding masses were calculated as per their mass/charge (m/Z) ratios. The fractions containing pure peptides were lyophilized and stored at −80 °C until further use.

### Fe–S Assembly

In vitro reconstitution was performed using established protocols with minor modifications.^46^ For in vitro reconstitution, 250 μM of apo-peptide in 50 mM Tris, pH 8.0, and 200 mM NaCl (buffer A) was taken inside a glovebox (Coy) and left for 1 h to make it completely anaerobic. All of the buffers used for reconstitution were previously purged with nitrogen and moved inside the anaerobic chamber. Before reconstituting with Fe–S clusters, the cysteines of the apopeptides were reduced by adding 5 mM dithiothreitol (DTT). After 30 min, a 10 molar excess of ammonium iron­(III) citrate (Sigma-Aldrich) from a 1 M stock was added slowly over 5 min. Ten min following the iron addition, a 10 molar excess of sodium sulfide (Sigma-Aldrich) was added slowly over 5 min. The chemical reconstitution was continued for about 3 h inside the glovebox, after which the resulting amber-colored holo-peptide solution was passed through PD-10 columns (GE) twice and stored at 4 °C or used for subsequent experiments.

### Size Exclusion Chromatography

Size exclusion chromatography (SEC) was performed anaerobically to determine the oligomeric nature of the reconstituted holo-peptides. For SEC, the sample was injected onto a Superdex 75 10/300 (GE) column using fast protein liquid chromatography (FPLC, Bio-Rad). The buffer B (10 mM Tris, pH 8.0, and 40 mM NaCl) was continuously purged with nitrogen gas throughout the run to maintain the anaerobicity. The chromatogram was monitored at both 280 nm (for protein) and 415 nm (for Fe–S cluster), respectively.

### UV–Vis Spectroscopy

Holopeptide samples were individually analyzed in 1 cm quartz cuvettes using a Cary 60 UV–vis spectrophotometer (Agilent Technologies).

### CD Spectroscopy

Thermal denaturation studies were conducted using CD spectroscopy on an AVIV 420 instrument. Each individual experiment was performed using 250 μL of 25 μM holoprotein/holopeptide in a 1 mm quartz cuvette, and the changes in ellipticity were monitored at 208 nm. The initial temperature was set to 20 °C, which was increased in 2 °C increments with an incubation period of 8 min at each step. Melting temperatures (*T*_1/2_) for all holoproteins and holopeptides, except for 2SC and SCC, were calculated using the classical Hill equation. For 2SC and SCC, a nonlinear fit (eq 1) described by Yadav et al. was employed individually to determine the melting temperatures.^102^

1 $$y\left(T\right)=\left\{\left(\mathrm{aN}+\mathrm{bN}\left(T\right)\right)+\left(\mathrm{aD}+\mathrm{bD}\left(T\right)\right)\exp\left[-\Delta H^{\mathrm{Van}}/R\left(\left[1/T\right]-\left[1/\mathrm{Tm}\right]\right)\right]\right\}/\left\{1+\exp\left[-\Delta H^{\mathrm{Van}}/R\left(\left[1/T\right]-\left[1/\mathrm{Tm}\right]\right)\right]\right\}$$

where a and b are two temperature-independent constants, N and D represent the native and denatured states of the protein, ΔH^Van^ corresponds to changes in enthalpy, R is the universal gas constant (1.9872 × 10^–3^ kcal K^–1^ mol^–1^), T is the temperature in Kelvin.

### EPR

For EPR spectroscopy, 200 μL of 100 μM holopeptide in buffer A, additionally supplemented with 20% glycerol as a cryoprotectant, was chemically reduced with 20 mM sodium dithionite (NaDt). The reduced sample was then transferred to a quartz tube and sealed anaerobically. CW-EPR spectra were recorded using an X-band Bruker EPR spectrometer (E580e) at 10 K, and the temperature was maintained using a helium-flow cryostat (Oxford ESR900). Other experimental parameters were 9.49 GHz microwave frequency, 0.2 mW microwave power, and 1 mT modulation amplitude.

### Potentiometric Titrations

All titrations were carried out anaerobically using a gold electrode (Pine Research) and a potentiostat (SP-50, BioLogic) to control the current flow, with UV–visible spectroscopy (Cary 60, Agilent Technologies) monitoring the reduction and oxidation of [4Fe-4S] clusters in the visible region. The midpoint potentials for each semidoxin have been determined by fitting the data points corresponding to 430 nm into the Nernst equation, as described in eq 2,^103,104^

2 $$\mathrm{pR}=1/\left(e^{\left[\left(E-\mathrm{Em}\right)/\left(RT/nF\right)\right]}\right)$$

where pR is the percentage reduced as a function of potential given by E, Em is the calculated midpoint potential in mV, R is the ideal gas constant, F is the Faraday constant, T is the temperature in Kelvin.

### Biological Electron Transfer Assay

Previously studied plasmids were used to perform biological electron transfer assays,^75^ including pSAC01, which constitutively expresses corn Fd-NADP reductase (FNR) and corn sulfite reductase (SIR), and pFd007, which expresses *Mastigocladus laminosus* Fd (mlFd1) using an aTc-inducible expression system. The latter plasmid was used as a positive control and to create vectors that express a nonfunctional mutant version of mlFd1 (mlFd1C42A), a symdoxin (pFdSCC), and all semidoxins (pFdAN, pFdAC, pFdSN, pFdSC). These vectors were built using Golden Gate Assembly.^105^ Ribosomal binding sites (RBS) were designed with high translation initiation rates using the RBS calculator.^106^ For nonselective growth, M9c cultures were prepared as described with one alteration,^75^ ferric citrate and MgSO_4_ concentrations at all growth phases were adjusted to 500 μM and 2 mM, respectively. Individual colonies were used to inoculate M9c liquid cultures in deep-well 96-well plates, 0.5 mL each, and cultures were incubated for 18 h at 37 °C while shaking at 300 rpm under aerobic conditions. To minimize residual sulfide, these cultures were centrifuged to pellet cells, and cells were resuspended in a mixture consisting of 100 μL supernatant and 900 μL of M9 selective medium (M9sa), which is identical to M9c but lacks cysteine and methionine.^75^ M9sa had ferric citrate and MgSO_4_ concentrations as M9c. To evaluate Fd electron transfer in cells, washed cultures were diluted ∼100-fold by using replicator pins to transfer 1 μL of culture into 100 μL of fresh M9sa. Cells were grown in the presence of the indicated aTc concentrations with the terminal electron acceptor (6 g/L trimethylamine N-oxide) in sterile Nunc Edge 2.0 96-well plates, with their side reservoirs filled with 1 mL of water. The plates were incubated in a Tecan Spark plate reader at 37 °C under the indicated O_2_ atmospheric concentrations while shaking at 300 rpm in double orbital mode.

### Structure Predictions

Ferredoxin models were generated using Boltz-1,^66^ a deep-learning-based open-source structure prediction platform that is general to proteins, nucleic acids, metal ions, and small molecules. [4Fe-4S] clusters were specified using the Chemical Component Dictionary code “SF4.”^107^ Boltz-1 recovers the structure of an experimentally determined ferredoxin to within 0.36 Cα Å RMSD (Boltz-1 model compared to 2FDN) and matches relative first-shell cysteine ligands and [4Fe-4S] cluster positions with the same accuracy (Figure S8). Sequences were provided in FASTA format. Symdoxins and full-length ferredoxins were modeled by using one protein chain and two [4Fe-4S] clusters. Semidoxin dimers were modeled by using two copies of the chain and two [4Fe-4S] clusters. Automatically generated multiple sequence alignments produced by ColabFold were used.^67^

### Phylogenetics

The ferredoxin signature from Interpro^80^ was used to scan for homologues in 7079 complete genomes. A total of 974 homologues were identified that were aligned using the MAFFT server with settings. Alignments were visualized in UGENE v.37.0 ^108^ to first ensure that halfway points generally matched in column in the alignment and did not separate any stretches of aligned residues between C- and N-termini. It was used to split each sequence into aligned C- and *N*- termini. Positions with >10% gaps in the alignment were then eliminated to remove extraneous gaps.

The multiple sequence alignment was used to reconstruct a maximum-likelihood phylogenetic tree using IQ-TREE v.1.6.12 software^109^ with 1000 Bootstrap iterations. ModelFinder^110^ in IQ-TREE was used to select the optimal model for tree construction, using the Bayesian Information Criterion (BIC) to balance model fit and simplicity. The model with the lowest BIC score was considered the best and hence was chosen to reconstruct the tree. Distances in terms of tree scale were calculated between paired C- and N- termini using the cophenetic.phylo function in R.^111^ Isoelectric points were calculated from sequences using the Bio.SeqUtils.IsoelectricPoint module.^112^

## Supplementary Material



## Notes

MS analysis of apo-semidoxins, oxygen sensitivity of [4Fe-4S]^2+^ resting state in 2AN and ANN; relative abundance of protodoxins; MS analysis of apo-PD1; UV–visible spectra of PD1; redox titrations on PD1; model of PD1 found inside a larger fold; superposition of the Boltz-1 model and the corresponding experimental structures; far-UV CD spectra for cluster-bound semidoxin and cognate symdoxins; and structural similarity of cognate semi- and symdoxins (PDF)
