# Supplementary material for: Designing Peptide Fossils That Model the Evolution of the Bacterial Ferredoxin Fold
Source: JACS Au. 2025 Nov 3;5(11):5414–26. doi: 10.1021/jacsau.5c00863 (PMC12648291; doi:10.1021/jacsau.5c00863)
Supplement: Supplementary file 1 [file au5c00863_si_001.pdf]

Supporting Information: Designing peptide fossils that model the evolution of the bacterial ferredoxin fold

AUTHORS: Bhanu P. Jagilinki[1,2], Ian Campbell[3], Alexei M. Tyryshkin[1,2], Andrew C. Mutter[1,2], Jan Siess[1], Juliana DiGiacomo[1], Dylan Klein[1], Saroj Poudel[1,2], Paul G. Falkowski[2], Jonathan J. Silberg[3], Vikas Nanda[1*]

AFFILIATIONS:

[1] Center for Advanced Biotechnology and Medicine and the Department of Biochemistry and Molecular Biology, Robert Wood Johnson Medical School, Rutgers University, Piscataway, NJ 08854, USA

[2] Environmental Biophysics and Molecular Ecology Program, Department of Marine and Coastal Sciences and the Department of Earth and Planetary Sciences, Rutgers University, New Brunswick, NJ 08901, USA

[3] Department of Biosciences, Rice University, Houston, TX 77005, USA

*correspondence: vik.nanda@rutgers.edu

Contents

	page
Fig. S1: MS analysis of apo-semidoxins	S3
Fig. S2: Oxygen sensitivity of [4Fe-4S]2+ resting state in 2AN and ANN	S4
Fig. S3: Relative abundance of protodoxins	S5
Fig. S4: MS analysis of apo-PD1	S6
Fig. S5: UV-visible spectra of PD1	S7
Fig. S6: Redox titrations on PD1	S8
Fig. S7: PD1 found inside larger protein	S9
Fig. S8:Superposition of Boltz-1 model and experimental structure	S10
Fig. S9:Far-UV CD spectra for cluster-bound semidoxin and cognate symdoxins	S11
Fig. S10: Structural similarity of cognate semi- and symdoxins	S12

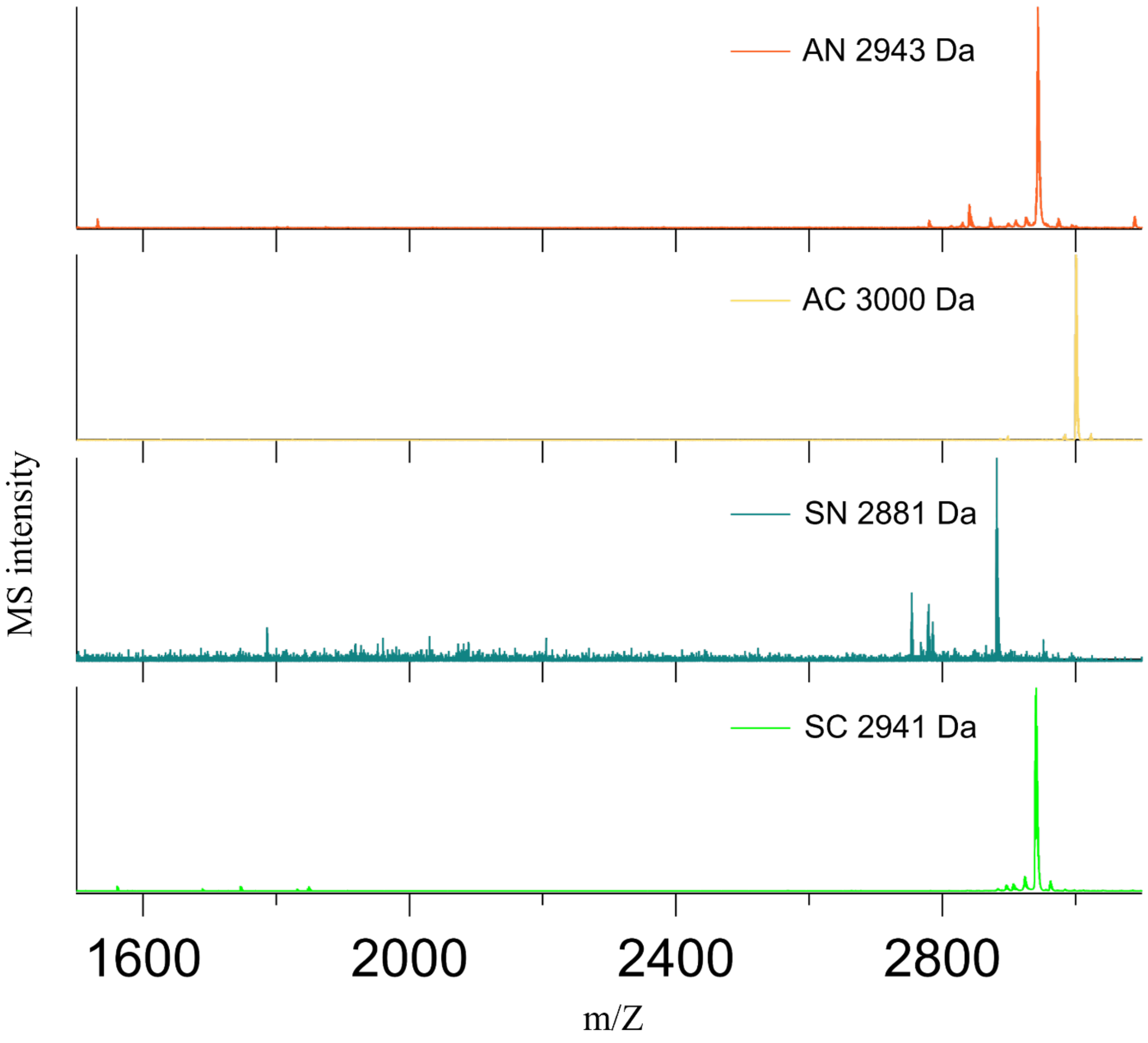

Fig. S1. MS analysis of apo-semidoxins. The MS spectra show the observed mass of apo-peptides for AN, AC, SN and SC respectively.

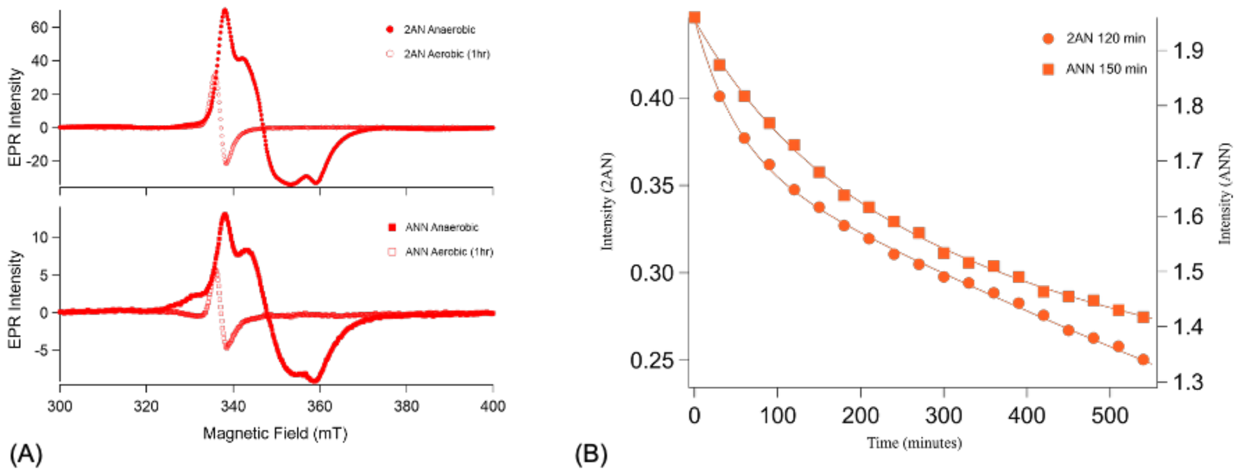

Fig. S2. Oxygen sensitivity of [4Fe-4S]$^{2+}$ resting state in 2AN and ANN. (A) EPR spectroscopy was used to elucidate the oxygen tolerance of the [4Fe-4S]$^{1+}$ cluster in its reduced state within 2AN and ANN. The EPR spectra reveal that the reduced [4Fe-4S]$^{1+}$ state is highly vulnerable to molecular oxygen in both 2AN and ANN. **(B)** UV-visible spectroscopy was used to elucidate the oxygen tolerance of the [4Fe-4S]$^{2+}$ cluster in its resting state within 2AN and ANN, and the changes corresponding to 433 nm on Y-axis was plotted against time (in minutes) on X-axis. The half-lives ($T_{1/2}$) have been determined as 120 minutes and 150 minutes respectively for 2AN and ANN.

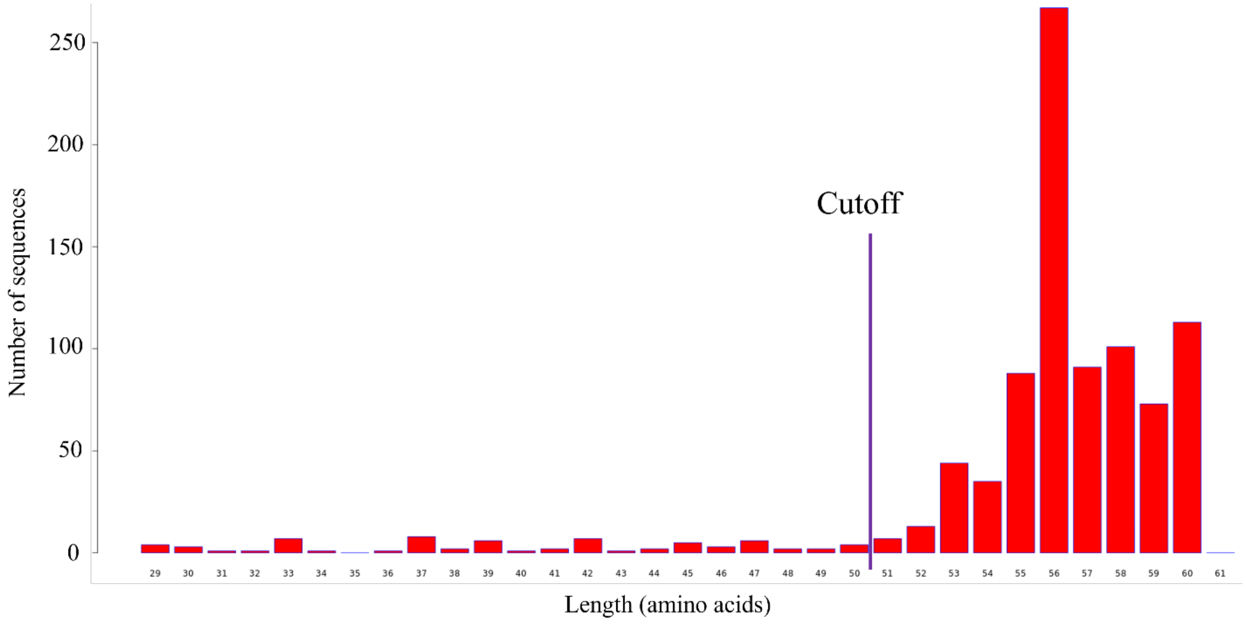

Fig. S3. Relative abundance of protodoxins. Naturally occurring semidoxin-like sequences form the existing databases. The cutoff used to identify potential sequences was set to 50, to eliminate the possibility of pooling ferredoxin like sequences.

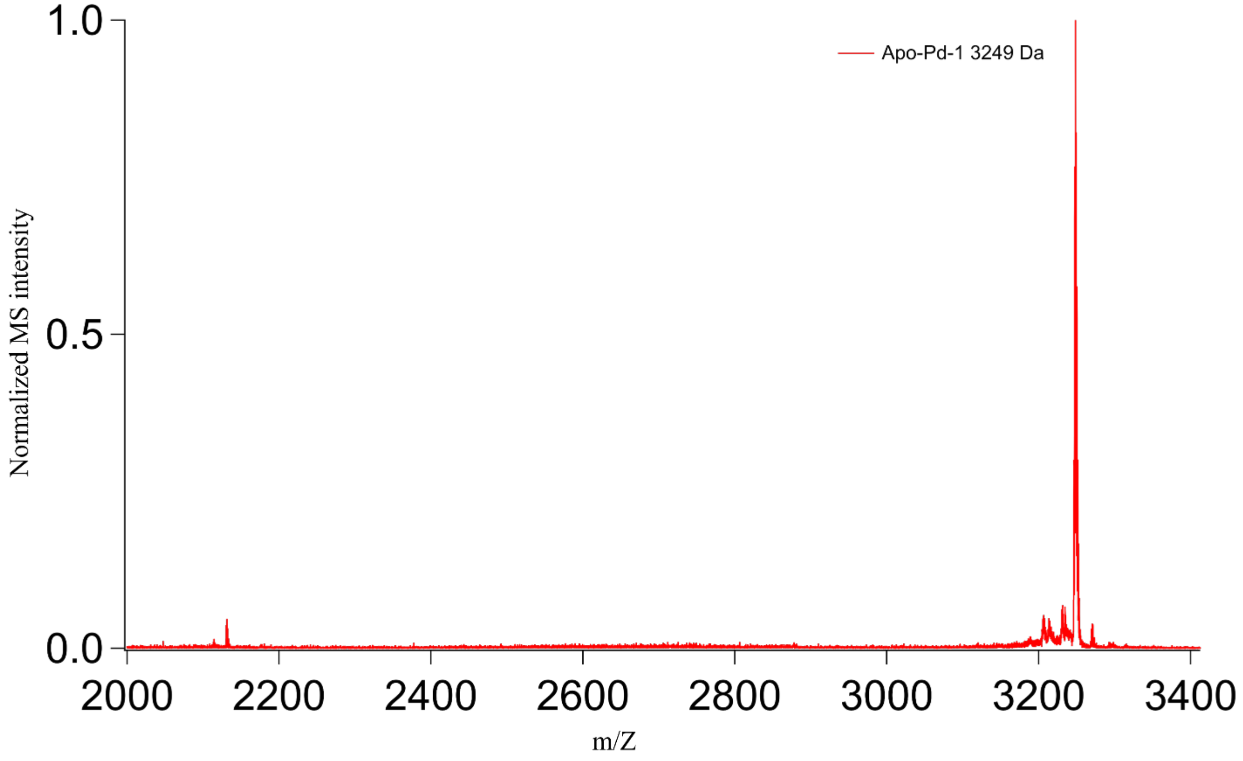

Fig. S4. MS analysis of apo-PD1. The mass of apo-PD1 is determined as 3249 Da, closely matching its theoretical mass.

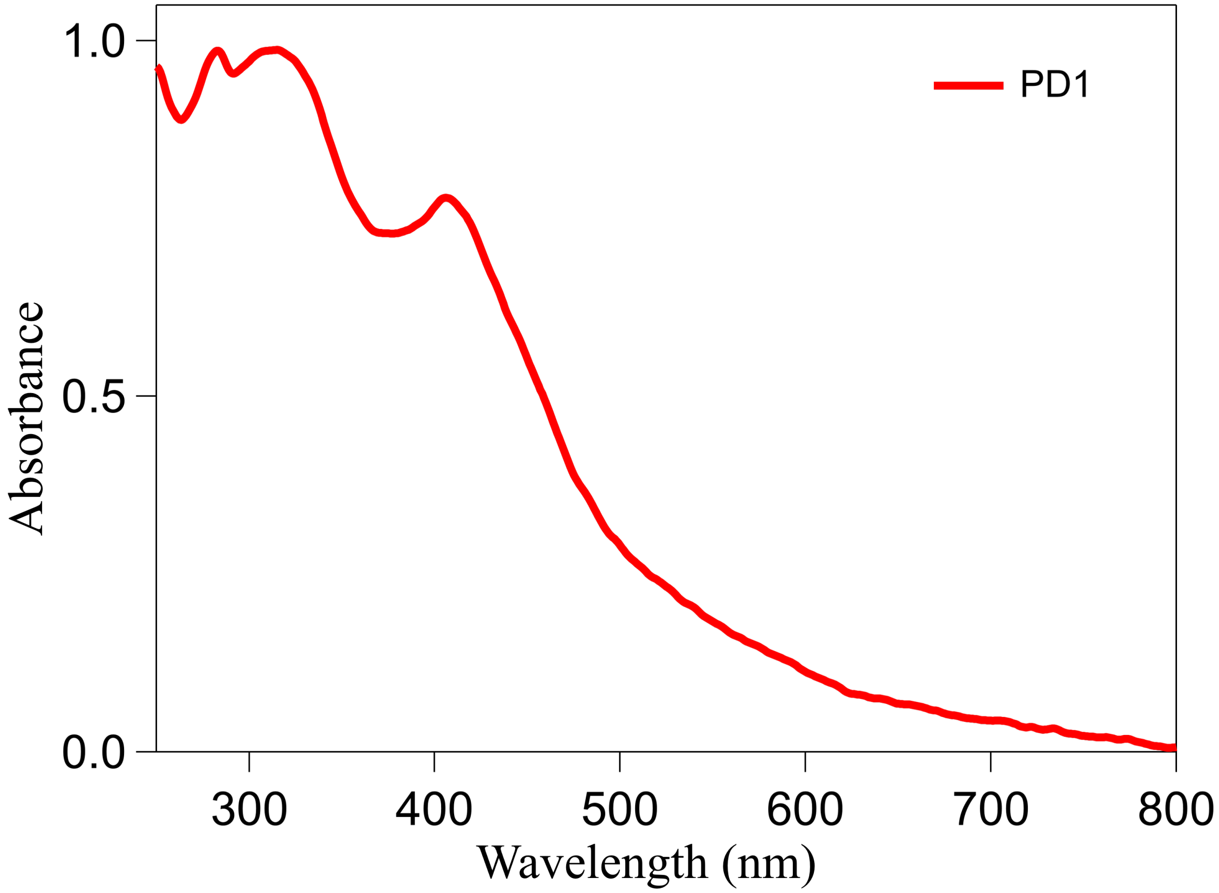

Fig. S5. UV-visible spectra of PD1. UV-visible spectra of PD1 displaying a characteristic 400 nm peak typical of Fe-S proteins.

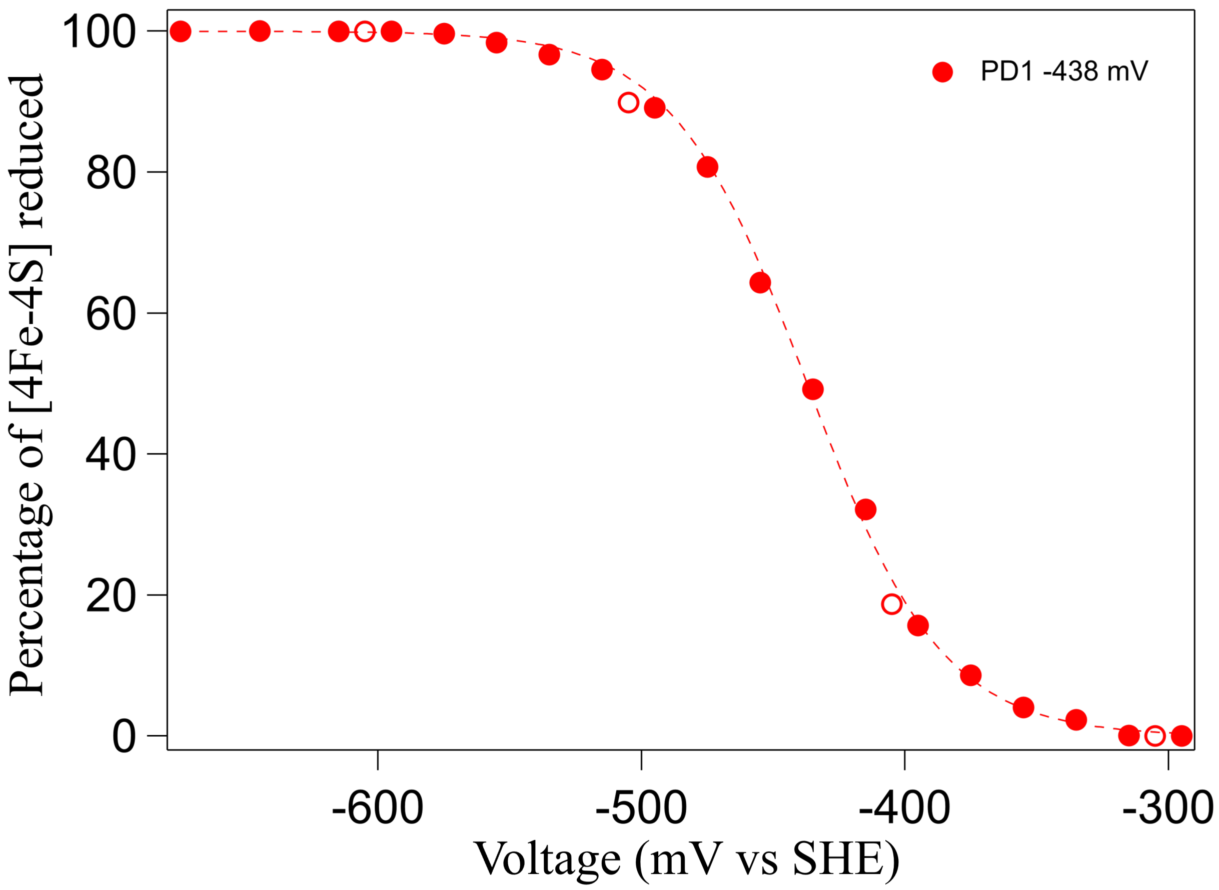

Fig. S6. Redox titrations on PD1. The electrochemical titration performed similarly to semidoxins, with the Y-axis representing percentage changes observed in UV spectra at 430 nm and the X-axis representing applied voltage (mV). The midpoint potential (Em) for PD1 was determined using the Nernst equation as -438 mV. Closed circles indicate the reduction cycle, while open circles denote the oxidation cycle.

Fig. S7 – PD1 found inside larger protein.

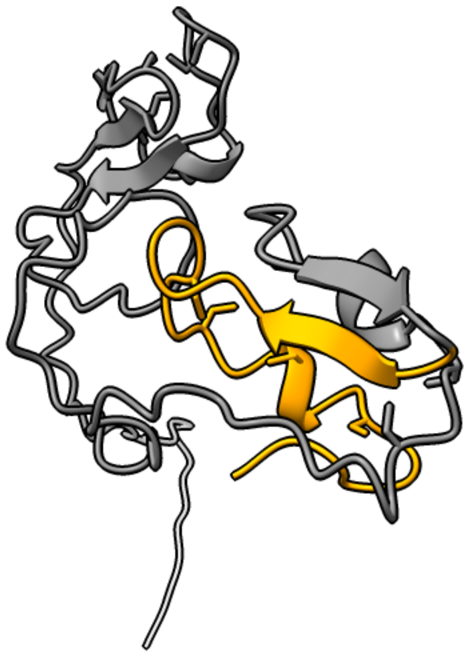

RefSeq: WP_019907887.1 4Fe-4S binding protein [Thermoanaerobacter indiensis]

```
MPGFGREFGGRGGRFGGGGGFGRGAGFGPDGSCVCENCGHEVPHQRGVPCYTLRCPVCGGPMIR
KAAGYGGTYRNSPTANKPVTEEKKDSNVKYTKPHKPKVDESACIGCGNCVRICPFNAIELKDGV
AYIDPKKCRDCGRCIDICPVGAIS
```

```
AIELKDGVAYIDPKKCRDCGRCIDICPVGAIS   A0A1I2A5A1
  ||.|.||||||||||||||||||||||||
 KLKNGIAYIDPKKCRDCGRCIDICPVGAIS    PD1
```

Figure S8: Superposition of Boltz-1 model (red) and experimental structure (blue – 2FDN) for *Clostridium acidiurici* ferredoxin.

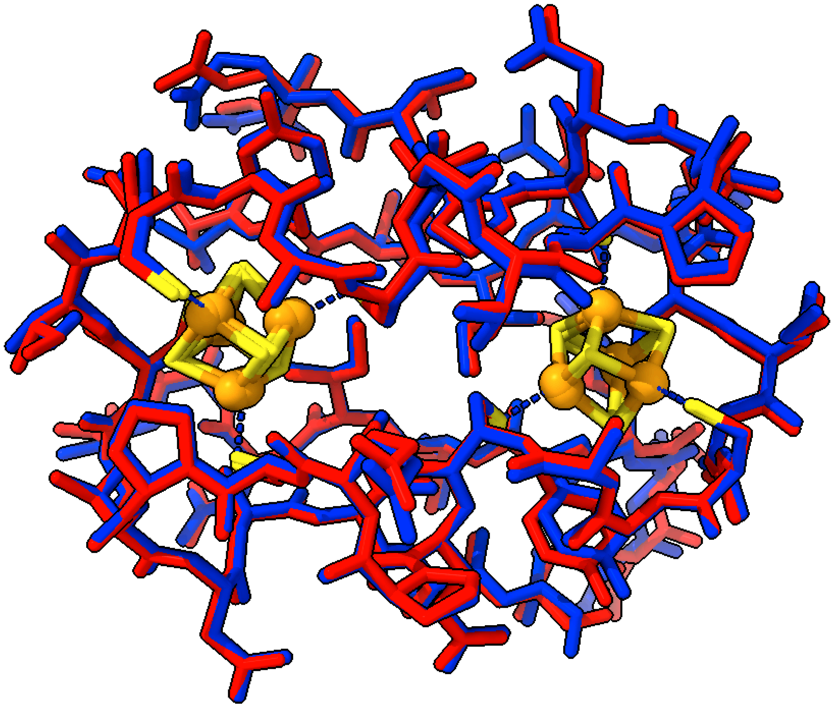

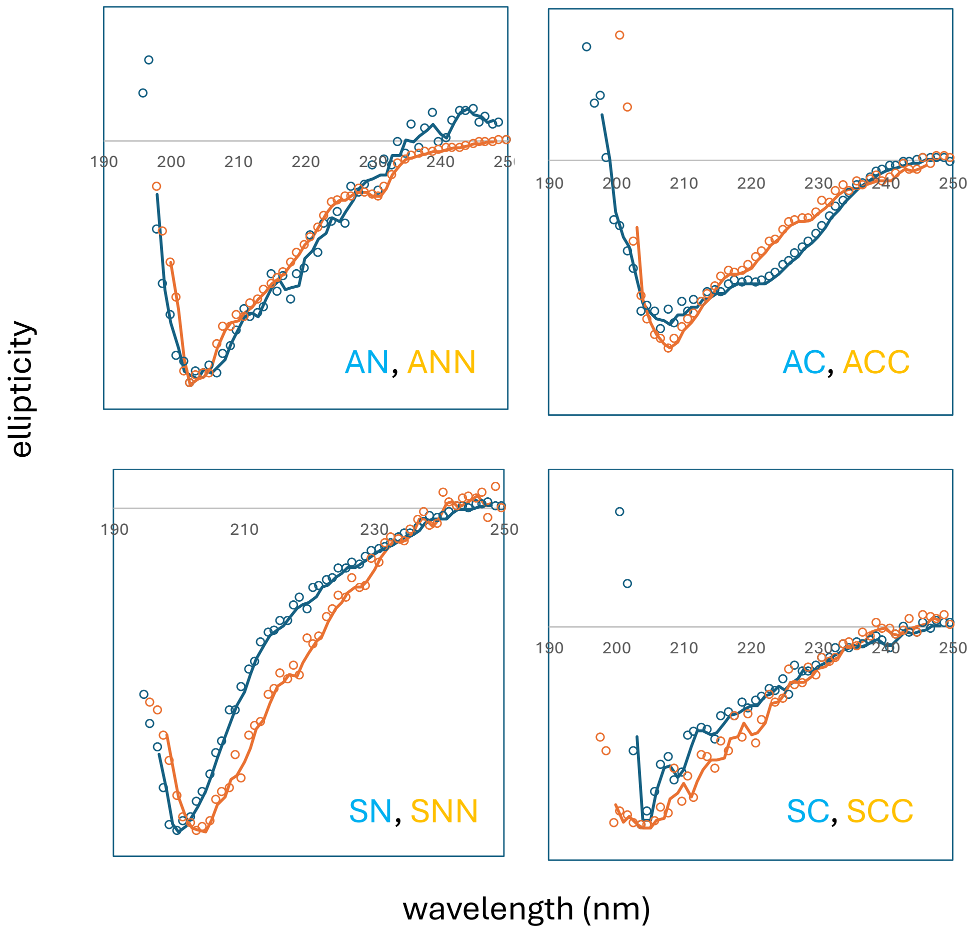

Figure S9: Far-UV CD spectra for cluster-bound semidoxin and cognate symdoxins. Spectra were collected at 25°C 50 mM Tris, pH 8.0, and 200 mM NaCl.

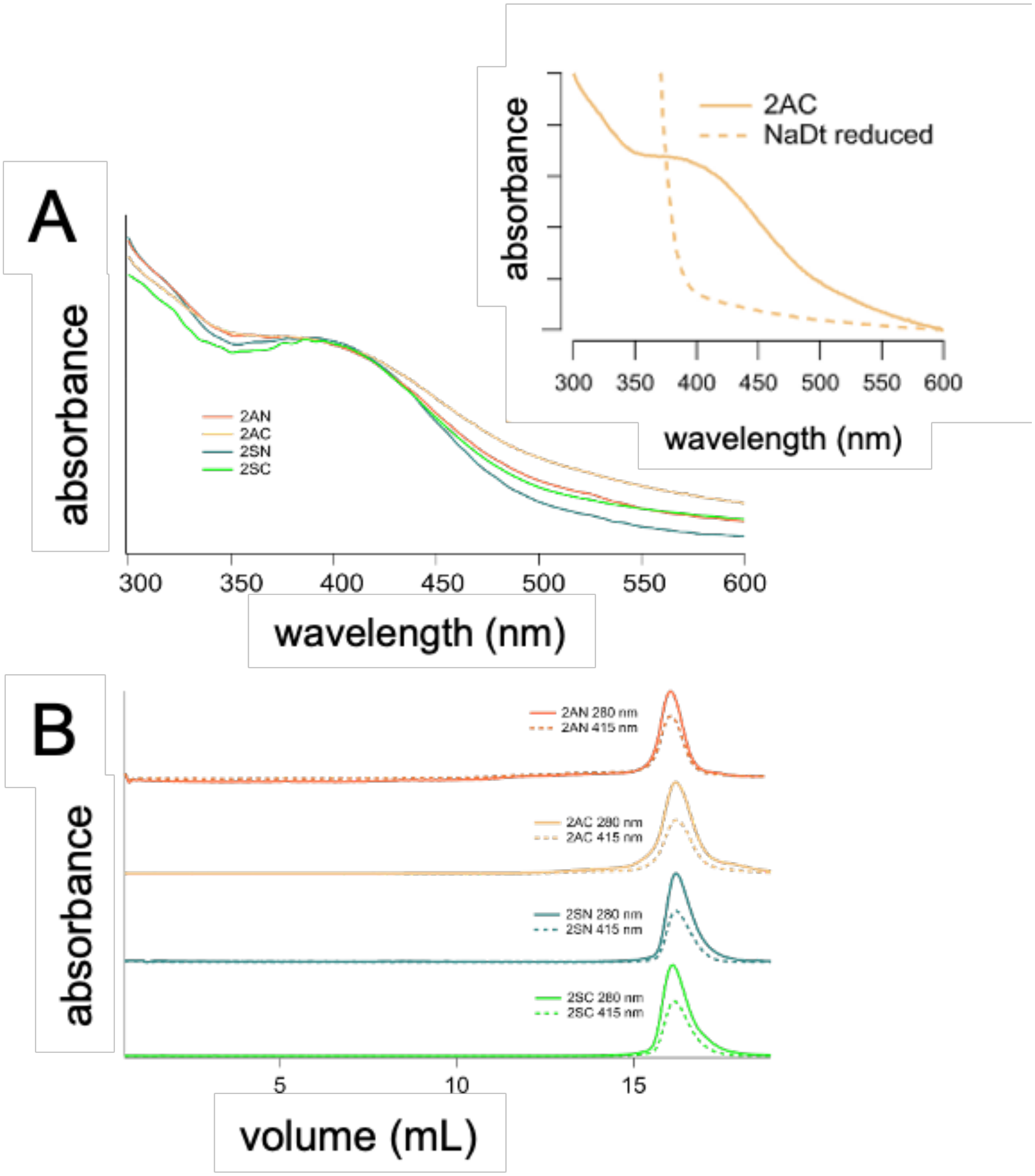

Figure S10: Structural similarity of cognate semi- and symdoxins. (**A**) UV-visible spectra of the four semidoxins exhibit a characteristic 400 nm peak indicative of iron-sulfur binding. Inset - Complete reduction of reconstituted 2AC (solid line) by addition of NaDT (broken lines). (**B**) Anaerobic FPLC chromatograms confirm a single dimeric species that binds the iron-sulfur cluster.
